# 

**Published:** 1867-07

**Authors:** 


### CONTENTS.

### ORIGINAL CONTRIBUTIONS.

ART. XXVIII. Concentrated Organic Remedies. By F. R. Payne, M.D., Marshall, Ill. ... 385

ART. XXIX. Abbreviated Report on Obstetrics. By Hiram Nance, M.D., Kewanee, Ill. ... 392

ART. XXX. Case of Hemorrhage and Abortion. By Dr. T. B. Trimble, M.D., Chicago ... 410

ART. XXXI. Valedictory Address. By R. E. McVey, M.D., President of the Morgan County Medical Society. (Delivered at the Anniversary Meeting, May 9th, 1867 ... 414

### PROCEEDINGS OF SOCIETIES.

Seventeenth Annual Meeting of the Illinois State Medical Society, Springfield, June 4th and 5th, 1867 ... 418

Election of Members ... 419

Nominating Committee, ... 420

Officers ... 421

Standing and Special Committees ... 425

Report of the Committee to Urge the Enactment of Suitable Laws by the State Legislature ... 428

Report of the Committee on Publication ... 429

Committees ... 432

Delegates to the American Medical Association ... 434

### SELECTIONS.

Case of Trephining the Spine; Death from Pyæmia; Clinical Remarks ... 437

### BOOK NOTICES.

Elements of Human Anatomy; General, Descriptive, and Practical. By T. G. Richardson, M.D., Prof. of Anatomy in the Medical Department of the University of Louisania. Second Edition. Carefully Revised and Illustrated by nearly 300 engravings. Philadelphia: J. P. Lippincott & Co. 1867 ... 439

Notes on the Origin, Nature, Prevention, and Treatment of Asiatic Cholera. By John C. Peters, M.D. Second Edition, with an Appendix. New York: D. Van Nostrand, 192 Broadway. 1867 ... 439

### EDITORIAL.

Illinois State Medical Society ... 440

Medical Education ... 440

Quarterly Journal of Psychological Medicine and Medical Jurisprudence ... 441

Obituary ... 441

Annual Announcement ... 442

Dr. Baker Brown of London ... 442

Wanted ... 442

Money Receipts from May 27th to June 26th ... 443

Mortality Report ... 443

